# Editorial: Algal symbiotic relationships in freshwater and marine environments

**DOI:** 10.3389/fpls.2023.1155759

**Published:** 2023-02-20

**Authors:** Leila Tirichine, Gwenael Piganeau

**Affiliations:** ^1^ Nantes Université, CNRS, US2B, UMR 6286, Nantes, France; ^2^ Integrative Biology of Marine Organisms (BIOM), Sorbonne University, CNRS, Oceanological Observatory of Banyuls, Banyuls-sur-Mer, France

**Keywords:** symbiosis, algae, bacteria, microbes, ecology, evolution, aquatic environments

Living organisms have never been solitary individuals and symbiotic relationships are challenging our very conception of the individual. Symbiosis, initially defined as a living together of different organisms (De Bary, 1879; Raval et al., 2022) represents a range of complex and intermingled relationships (mutualism, commensalism amensalism and parasitism). Although actively debated with numerous theories on the origin of eukaryotes, it is widely accepted that a metabolic symbiosis and successive endosymbioses were important in the evolution of eukaryotes and their diversification (Hartman and Fedorov, 2002; Sapp, 2004; Embley and Martin, 2006; Cenci et al., 2017; Sibbald and Archibald, 2020; Gabaldon, 2021). Thus, symbiosis conceptually challenges our view of the processes of evolution, beyond mutation, recombination and natural selection (Margulis and Fester, 1991). The most studied symbioses involve complex eukaryotes and microorganisms in both terrestrial and aquatic habitats (Bais et al., 2006; The Human Microbiome Project Consortium, 2012; Blackall LLW, 2015), but a growing number of studies reveal ubiquitous symbioses among microscopic life forms (Decelle et al., 2012; Foster and Zehr, 2019). With its diversity and outcomes combining metabolic capabilities of interacting partners, symbiosis is recognized as the most important evolutionary process that has allowed the appearance of new genomes/species throughout the history of life on Earth (Kiers and West, 2015; O’Malley, 2015).

Some symbioses are key to the existence of entire ecosystems such as the interactions underpinning the success of coral reefs, the cnidarian-algae mutualism that provide habitat for roughly one fourth of all marine life (Blackall LLW, 2015; Frankowiak et al., 2016). In this symbiosis, metabolites from the animal host (corals, sea anemones, jellyfish, and hydrocorals) are exchanged for microalgal exudates (Davy et al., 2012). Another symbiotic interaction that deserves greater attention in microbial networks is between parasitic fungi and phytoplankton as it has a significant impact on ecosystem functioning. These symbiotic interactions cause the transfer of photosynthetic carbon to infecting fungi and the stimulation of bacterial colonization on phytoplankton cells, altering ultimately bacterial community composition and subsequently carbon flow (Tourneroche et al., 2019; Klawonn et al., 2021). Although artificial, a beneficial symbiotic interaction was reported between the freshwater chlorophyte, *Chlamydomonas reinhardtii* and diverse ascomycete fungi that provide nitrogen to the algae (Simon et al., 2017). Recently, similar artificial symbiosis was reported in the marine microalgae *Nannochloropsis oceanica* and a terrestrial fungus, *Mortierella elongate.* Here, an unusual interaction takes place as functional algal cells are included within fungal mycelium, while in all known algae-fungus interactions, the algal cells remained external to fungal hyphae (Du et al., 2018; Du et al., 2019). This study showed the stability of the interaction with a bidirectional exchange of nutrients suggesting this could be the beginning of an endosymbiogenesis within eukaryotes.

Despite this recent recognition and striking importance, symbiosis received less attention compared to other fields of investigation and remains largely unexplored in particular in aquatic biota where the nature of the environment, fluid, represents an additional challenge. The advent of symbiosis as a critical area of research is challenged by the difficulties in maintaining symbiotic partners/holobiont alive in lab cultures, in particular for obligate interactions when both partners are dependent on each other for survival. Often, commonly used culturing techniques are not suitable to species in symbiotic relationships. The lack of knowledge about their genome background and metabolic capabilities, to predict their nutrient requirements and culture conditions, hinders the progress in this field. However, recent and rapid advances in whole genome sequencing of either individual species and/or meta-communities helped to overcome some of these bottlenecks, using data mining for designing custom based media that fulfill the needs of symbiotic organisms (Leon et al., 2014; Jaswal et al., 2019; Lugli et al., 2019). Another fundamental boost is undoubtedly the existence of established model organisms that open up novel avenues of investigations, which would otherwise be impossible or at least difficult to achieve. A recent study that used both metagenomics sequencing and a model species, the diatom *Phaeodactylum tricornutum*, successfully identified an overlooked symbiosis between microalgae and non-cyanobacteria diazotrophs (NCDs) which challenges the long-held paradigm of dominance of cyanobacteria interactions with microalgae over NCDs and brings the first hints on how heterotrophic proteobacteria thrive in surface waters and oxygenated areas (Chandola et al., 2022).

The articles included in this Research Topic provide a view of how symbiotic interactions can help bypass environmental stresses such as in Heo et al., where filamentous ascomycetes *Arthrinium* species act as endosymbionts protecting brown algae from oxidative stress. Miao et al. studied the diversity and function of gammarid like animals that feed on the blooming green tides of *Ulva prolifera* contributing to their containment and limiting their negative impact on the environment. Lo et al. address the methodological challenge of inferring phylogenetic relationships from large genome assemblies in the (1-5Gb) genomes of Dinoflagellates *Symbodinium*, essential symbionts of corals. Their *in silico* analyses demonstrate that a scalable k-mer approach largely agrees with the phylogenetic signal inferred from the LSU rDNA sequence. The combination of genomic and experimental data sometimes allows us to hypothesize about the metabolic bases of coexistence, as in the case of coexistence between *Roseovarius* and the green alga *Ostreococcus tauri*, where the bacterial genomes encodes the metabolic pathway to produce the vitamins needed by the microalgae (Vacant et al). This study reports a stable coexistence maintaining the microalgae and the bacterium over several years, unlike the dynamic associations reported between *Dinoroseobacter shibae* and the microalgae *Prorocebtrum minimum* (Mansky et al., 2021), or Sulfitobacter and *Emiliania huxleyi* (Barak-Gavish et al., 2023).

The field of symbiosis in aquatic habitats is expanding quickly with both *in silico* and experimental approaches, and many novel insights into species interactions, their ecology and evolution are expected in the near future.

## Author contributions

All authors listed have made a substantial, direct, and intellectual contribution to the work and approved it for publication.
